# Outpatient Foley catheter versus inpatient prostaglandin E2 gel for induction of labour: a randomised trial

**DOI:** 10.1186/1471-2393-13-25

**Published:** 2013-01-29

**Authors:** Amanda Henry, Arushi Madan, Rachel Reid, Sally K Tracy, Kathryn Austin, Alec Welsh, Daniel Challis

**Affiliations:** 1 School of Women’s and Children’s Health, University of New South Wales, Kensington, Australia; 2 Department of Maternal-Fetal Medicine, Royal Hospital for Women, Sydney, Australia; 3 Midwifery and Women’s Health Research Unit, University of Sydney, Sydney, Australia

**Keywords:** Induction of labour, Mechanical ripening, Prostaglandin, Foley catheter, Randomised controlled trial, Unfavourable cervix

## Abstract

**Background:**

Induction of labour (IOL) is one of the commonest obstetric interventions, with significant impact on both the individual woman and health service delivery. Outpatient IOL is an attractive option to reduce these impacts. To date there is little data comparing outpatient and inpatient IOL methods, and potential safety concerns (hyperstimulation) if prostaglandins, the standard inpatient IOL medications, are used in the outpatient setting. The purpose of this study was to assess feasibility, clinical effectiveness and patient acceptability of outpatient Foley catheter (OPC) vs. inpatient vaginal PGE2 (IP) for induction of labour (IOL) at term.

**Methods:**

Women with an unfavourable cervix requiring IOL at term (N = 101) were randomised to outpatient care using Foley catheter (OPC, n = 50) or inpatient care using vaginal PGE2 (IP, n = 51). OPC group had Foley catheter inserted and were discharged overnight following a reassuring cardiotocograph. IP group received 2 mg/1 mg vaginal PGE2 if nulliparous or 1 mg/1 mg if multiparous. Main outcome measures were inpatient stay (prior to birth, in Birthing Unit, total), mode of birth, induction to delivery interval, adverse reactions and patient satisfaction.

**Results:**

OPC group had shorter hospital stay prior to birth (21.3 vs. 32.4 hrs, p < .001), IP were more likely to achieve vaginal birth within 12 hours of presenting to Birthing Unit (53% vs. 28%, p = .01). Vaginal birth rates (66% OPC Vs. 71% IP), total induction to delivery time (33.5 hrs vs. 31.3 hrs) and total inpatient times (96 hrs OPC Vs. 105 hrs IP) were similar. OPC group felt less pain (significant discomfort 26% Vs 58%, p = .003), and had more sleep (5.8 Vs 3.4 hours, p < .001), during cervical preparation, but were more likely to require oxytocin IOL (88 Vs 59%, p = .001).

**Conclusions:**

OPC was feasible and acceptable for IOL of women with an unfavourable cervix at term compared to IP, however did not show a statistically significant reduction in total inpatient stay and was associated with increased oxytocin IOL.

**Trial registration:**

Australian New Zealand Clinical Trials Registry, ACTRN:12609000420246.

## Background

Induction of labour (IOL) is one of the commonest obstetric interventions, occurring in approximately 25% of term pregnancies in developed countries [1]. For women with an unfavourable cervix requiring IOL, cervical preparation is usually recommended, as oxytocin use alone leads to a longer induction to delivery interval and possibly increased intervention [2]. Both chemical and mechanical methods for cervical preparation are available, with prostaglandin preparations (PGE1 and PGE2) used as the chemical method, and variations of intracervical catheter (either single or double balloon) the most widely studied mechanical method. Mechanical methods are used to dilate the cervix, but may also increase prostaglandin and/or oxytocin release by causing localised inflammation [3], while prostaglandin preparations act to promote both cervical remodelling and uterine activity [4].

The Cochrane review of mechanical methods of induction of labour [3] includes 71 randomised controlled trials (9722 women) and suggests mechanical methods have equivalent clinical effectiveness to prostaglandins (with no overall significant difference in Caesarean Section rates, vaginal delivery within 24 hours of induction, or need for oxytocin), and lower rates of hyperstimulation with fetal heart rate (FHR) changes compared to vaginal PGE2 (RR 0.16, 95% CI 0.06-0.39) and misoprostol (RR 0.37, 95% CI 0.25-0.54). In the review’s subgroup of balloon catheter vs. prostaglandins (23 studies, 3474 women) Caesarean Section, instrumental delivery rates and vaginal delivery in <24 hours did not differ significantly, and hyperstimulation with FHR changes was less with balloon catheter, however oxytocin augmentation was more likely in the catheter group (RR 1.51, 95% CI 1.15-1.97)[3]. Only one trial reported on patient satisfaction [5].

The concept of outpatient IOL, where cervical preparation and/or early labour occurs predominantly at home, is an attractive alternative to inpatient management both economically and for patient satisfaction. When compared to placebo or no treatment, outpatient induction using a variety of methods appears feasible and important adverse events are rare [6]. However only, 3 randomised studies (612 women total) comparing outpatient to inpatient IOL (active treatment in both arms) have previously been published, and do not demonstrate significant differences in clinical outcomes [7]. Of these studies, one randomised 111 women to outpatient versus inpatient single-balloon (Foley) catheter, finding no difference in clinical efficacy and an average decreased length of stay of 9.6 hours in the outpatient group [8].

Existing data suggests non-inferiority of single-balloon catheter versus PGE2 with regards to mode of birth and induction to delivery interval. In our hospital’s setting, inpatient balloon catheter is used when PGE2 is considered unsafe for cervical ripening e.g. previous Caesarean Section, but only medical staff are trained in its insertion, and inpatient PGE2 remains the standard cervical ripening method due to ease of administration for both midwifery and medical staff. Outpatient PGE2 is not used due to concerns about safety, particularly hyperstimulation [3]. We therefore aimed to investigate the use of Foley (single balloon) catheter for outpatient cervical preparation for its likely safety for outpatient use given low rates of excessive uterine activity, and potential resource and patient benefits of outpatient IOL. We were not aware of prior published research directly comparing outpatient catheter with inpatient PGE2 gel. We undertook a randomised trial to determine the feasibility, clinical effectiveness and acceptability to women of using intracervical Foley catheter in an outpatient setting vs. intravaginal Prostaglandin E2 (Prostin) gel in an inpatient setting for induction of labour (IOL).

## Methods

A non-blinded, randomised trial was performed between June 2009 and December 2010 at an Australian metropolitan tertiary teaching hospital with approximately 4200 births/year. Prior to the trial, inpatient IOL, using vaginal PGE2 for cervical preparation when required (Bishop Score <7 and cervical dilation <2 cm), or Foley catheter if there were contraindications to prostaglandin use, was standard care at the trial hospital. At this hospital, women booked for cervical preparation are admitted to the antenatal ward on their scheduled day, fetal and maternal assessment including vaginal examination (VE) occurs, then PGE2 is given or catheter inserted. Women are transferred to the Birthing Unit, the hospital’s high-acuity Labour and Delivery ward with increased midwifery and medical staffing ratios, the following morning if the cervix becomes favourable for ARM, or sooner if labour occurs or there are maternal or fetal concerns requiring continuous monitoring. If the cervix remains unfavourable the morning following cervical ripening, the patient’s clinician decides upon further management (usually either further PGE2 or insertion of Foley catheter).

During the trial, outpatient Foley catheter induction was also offered, within the trial setting only. Information pamphlets about the trial were available in the hospital’s antenatal clinic, and hospital staff requested to provide information about the trial and ensure patients were given a copy of the trial pamphlet at the time of IOL booking. Women were then screened for trial inclusion on arrival at the antenatal ward. Inclusion criteria were women ≥18 years old with a gestational age of 37 weeks or more, requiring IOL with a cervical preparation procedure (Bishop Score <7 and cervical dilation <2 cm). Prior to recruitment and randomisation, all eligible women underwent a baseline cardiotocograph (CTG) and VE to record the Bishop score/confirm eligibility. Exclusion criteria were:

1) Unsuitable for outpatient management

2) Unsuitable for randomisation to either PGE2 (e.g. previous Caesarean section) or catheter use (e.g. latex allergy), or prior attempted IOL in this pregnancy

3) No longer requiring cervical preparation (Bishop score ≥7 or cervical dilatation ≥2 cm), had ruptured membranes, or evidence of regular uterine contractions at time of booked induction

4) Multiple pregnancy or non-vertex presentation

5) Unable to give informed consent (e.g. secondary to insufficient English), or consent was declined

Eligible consenting women were then enrolled in the trial by the study investigators or the antenatal ward midwives, and randomised to either the outpatient Foley catheter or inpatient PGE2 gel arm (control group). Simple randomisation using a random number table was performed prior to trial commencement for 240 patients (50% allocated to OPC and 50% to IP). The allocation assignment was sealed in sequentially numbered, opaque envelopes by an individual not otherwise involved in the conduct of the trial. Envelopes were kept in a locked box in the Antenatal ward with keys held by midwifery staff. When trial consent was signed the next sequential envelope was removed from the box and opened to determine allocation. Participants, staff, and outcome assessors were not blinded to group assignment.

For women randomised to outpatient Foley catheter (OPC), speculum examination was performed by a resident trained in cervical catheter insertion, and a 16 F standard latex Foley catheter was inserted using aseptic technique above the internal cervical os and inflated with 30 mL of sterile water. The catheter was taped to the inner thigh with slight traction, and spigot inserted to occlude the lumen. A post-procedure CTG was performed for at least 30 minutes. When the CTG was reassuring, women were discharged home after counselling regarding possible discomfort, available pain relief, probability of catheter falling out, possibility of labour, and with written information and instructions regarding the catheter. They were also given analgesia (1 g paracetamol/60 mg codeine) and sedation (20 mg temazepam) to take home with instructions for use if required. Women were asked to return to the Birthing Unit at 7 AM the following morning, unless labour occurred or if they had any concerns.

Women randomised to inpatient PGE2 gel (IP), received the hospital’s normal PGE2 protocol of initial 2 mg dose PV for nulliparous and 1 mg PV for parous women, inserted into the posterior vaginal fornix. A post-insertion CTG was performed for at least 30 minutes. The cervix was re-examined after six hours and, if required, the procedure repeated using a further 1 mg PGE2 (regardless of parity). Analgesia (1 g paracetamol/60 mg codeine) and sedation (20 mg temazepam) were charted, and administered if required. As per hospital protocol, IP women were transferred to the Birthing Unit at 7 am the morning after PGE2 administration, unless ARM could not be performed, labour occurred prior (regular contractions +/− spontaneous rupture of membranes, and evidence of cervical change on VE), or there were maternal or fetal concerns on the ward.

If the cervix was still unfavourable or artificial rupture of membranes (ARM) not possible the morning following the start of cervical preparation, further clinical management was continued as decided by the patient and treating clinician. (For the IP group either further PGE2 was given or Foley catheter inserted, and the patient remained on Antenatal Ward until in labour, for the OPC group admission to Antenatal Ward for inpatient PGE2 occurred). Alternatively ARM was performed if possible and oxytocin infusion commenced as per hospital protocol.

### Data collection and statistical methods

Demographic data was collected at time of randomisation via direct patient questioning, while data on pregnancy, labour, birth and neonatal outcomes was collected using patient records and hospital databases. Women were followed-up and satisfaction surveys (Additional file 1) administered on the Postnatal Ward 24–48 hours post-birth. The pre-specified primary outcomes were 1) percentage of women delivering vaginally within 12 hours of transfer to the Birthing Unit and 2) total inpatient hours from time of randomisation to delivery. These are not typical IOL primary outcomes, as this was an inpatient vs. outpatient study and we considered total induction to vaginal delivery interval less critical. As admission/transfer to Birthing Unit was expected around 7 AM for both groups, birth within the following 12 hours (by early evening, avoiding overnight hours in this high-acuity, high-cost setting) was thought likely to be important to feasibility assessment of OPC. Pre-specified secondary outcomes considered clinical effectiveness, patient acceptability, and safety. For clinical effectiveness, outcomes included mode of birth, induction to delivery interval, vaginal delivery within 24 hours of commencement of cervical ripening, percentage of women requiring oxytocin, and total inpatient stay. Patient acceptability was assessed through the patient satisfaction questionnaire, and rate of unplanned (not in labour) hospital readmission. Safety was assessed through maternal or neonatal febrile morbidity (T 38.0+ degrees Celsius on 2+ occasions, or 38.5+ degrees Celsius once), non-reassuring FHR traces [9], operative delivery for fetal distress, Apgar scores, cord arterial pH (when available), and admission to newborn care. We examined nulliparous and parous women separately in a pre-specified subgroup analysis. Hyperstimulation was defined as 5 or more uterine contractions in 10 minutes associated with non-reassuring fetal heart rate pattern.

Initial sample size of 200 was based on an ability to detect with 80% power a 20% difference between groups for primary outcome 1, with a more modest sample of 96 required to detect (with 90% power) a 10 hour decrease in randomisation to delivery inpatient stay from 25 to 15 hours (SD 15 hours) [10]. Based on previous hospital IOL data and participation rate of 50% for screened women, time expected for recruitment was 9–12 months. The study’s Steering Group (AH, ST, DC, AM) reviewed results after 50 participants and extended recruitment by six months, however staffing and funding considerations precluded any subsequent extension and final sample was 101.

The database was maintained with Microsoft Excel and data analysed using SPSS (SPSS Statistics version 19.0, IBM corporation). Categorical data are presented as frequencies and percentages and analysed using the Chi-square test. Continuous variables are presented as means with standard deviation or medians with ranges and analysed using Student’s *t*-test and Mann–Whitney *U* test as appropriate for normally distributed and skewed data respectively. All tests are two-tailed with statistical significance defined as a probability value of <0.05. All data were analysed on an intention-to-treat basis.

Ethical approval was obtained from the Human Research Ethics Committee of the local Area Health Service and the University of New South Wales. The trial was prospectively registered in the Australasian Clinical Trials Registry, ACTRN:12609000420246.

## Results

**Figure 1:**
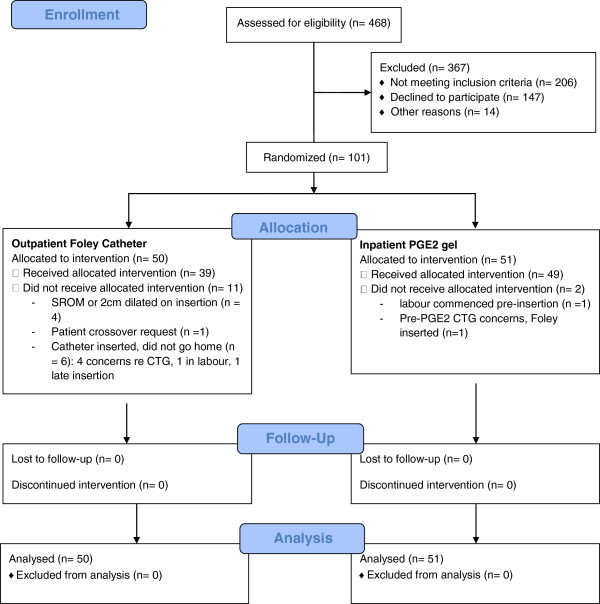
Participant flow diagram (CONSORT 2010).

Between June 2009 and December 2010, 468 women were screened for trial inclusion and 101 women were randomised, 50 women to OPC and 51 women to IP (Figure 1). Demographic data are summarised in Table 1. Women were well matched at trial entry, and, reflective of the hospital’s socioeconomically advantaged catchment, were predominantly aged over 30 and nulliparous. Pregnancy and induction data are summarised in Table 2. Most inductions were performed for post-dates, and over 80% of included women had a Bishop score of 4 or less.

**Table 1:** Baseline characteristics of study participants

**Baseline Characteristics at time of induction**	**Foley Catheter n (%) (Total n = 50)**	**PGE2 Gel n (%) (Total n = 51)**	**P-value**
Mean Age (years)	32.74	32.90	.865
Marital Status			
· Married/Defacto	49 (98)	50 (98)	.989
· Single/other	1 (2)	1 (2)	
Health Insurance Status			
· Public	49 (98)	48 (94)	.317
· Private	1 (2)	3 (6)	
Country of birth			.596
· Australia	27 (54)	21 (41)	
· UK/Europe	11 (22)	16 (31)	
· Asia-Pacific	8 (16)	10 (20)	
· Other	4 (8)	4 (8)	
Usual Employment			.758
· Manager	9 (18)	6 (12)	
· Professional	24 (48)	22 (43)	
· Clerical	6 (12)	7 (14)	
· Other	8 (16)	12 (24)	
· Unknown	3 (6)	4 (8)	
Parity			
· Nulliparous	45 (90)	46 (90)	.750
· Multiparous	5 (10)	5 (10)	
Smoking status			
· Ever smoker	10 (20)	16 (31)	.191
· Current smoker	1 (2)	2 (4)	
Past history			
· Any past medical	23 (46)	25 (49)	.761
· Any past surgical	30 (60)	22 (43)	.09
BMI (kg/m^2^)	24.1	23.0	.219
Model of care			.945
· Medical (ANC/GP/Private)	14 (28)	14 (27)	
· Midwifery clinic	22 (44)	24 (47)	
· Midwifery group practice	14 (28)	13 (26)	

**Table 2:** Induction characteristics of study participants

	**Foley Catheter n (%) (Total n = 50)**	**PGE2 Gel n (%) (Total n = 51)**	**P-value**
Gestational Age (weeks)	40.8	40.6	.521
Complications of pregnancy			.927
· None	28 (56)	30 (59)	
· Hypertensive disease	3 (6)	4 (8)	
· GDM	5 (10)	5 (10)	
· Other	13 (26)	10 (20)	
· Unknown	1 (2)	2 (4)	
Indication for Induction			.678
· Post-dates	38 (76)	35 (69)	
· Maternal Medical Concerns			
◦ Hypertensive Disease	3 (6)	2 (4)	
◦ Gestational Diabetes	3 (6)	6 (12)	
◦ Cholestasis	3 (6)	1 (2)	
· Fetal Concerns	1 (2)	3 (6)	
· Maternal Age	2 (4)	3 (6)	
· Social/Other	0 (0)	1 (2)	
GBS positive	7 (14)	7 (14)	.968
Preferred delivery area:			.425
· Delivery Suite	44 (88)	42 (82)	
· Birth Centre	6 (12)	9 (18)	
Cervical Sweep Performed	23 (46)	22 (43)	.772
Natural Ripening Methods tried (multiple responses)	26 (52)	28 (55)	.624
· Sexual intercourse	14 (28)	17 (33)	
· Herbal preparations	13 (26)	11 (22)	
· Acupuncture	11 (22)	11 (22)	
· Food/spices	4 (8)	4 (8)	
· Exercise/walking	9 (18)	11 (22)	
· Nipple/breast stimulation	4 (8)	2 (4)	
· Other	6 (12)	5 (10)	
Baseline Bishop Score	2.7 ± (1.7)	2.9 ± (1.7)	.643
Score category:			.945
· 0–2	23 (46)	20 (39)	
· 3–4	17 (34)	22 (43)	
· 5–6	8 (16)	9 (18)	

**Figure 2:**
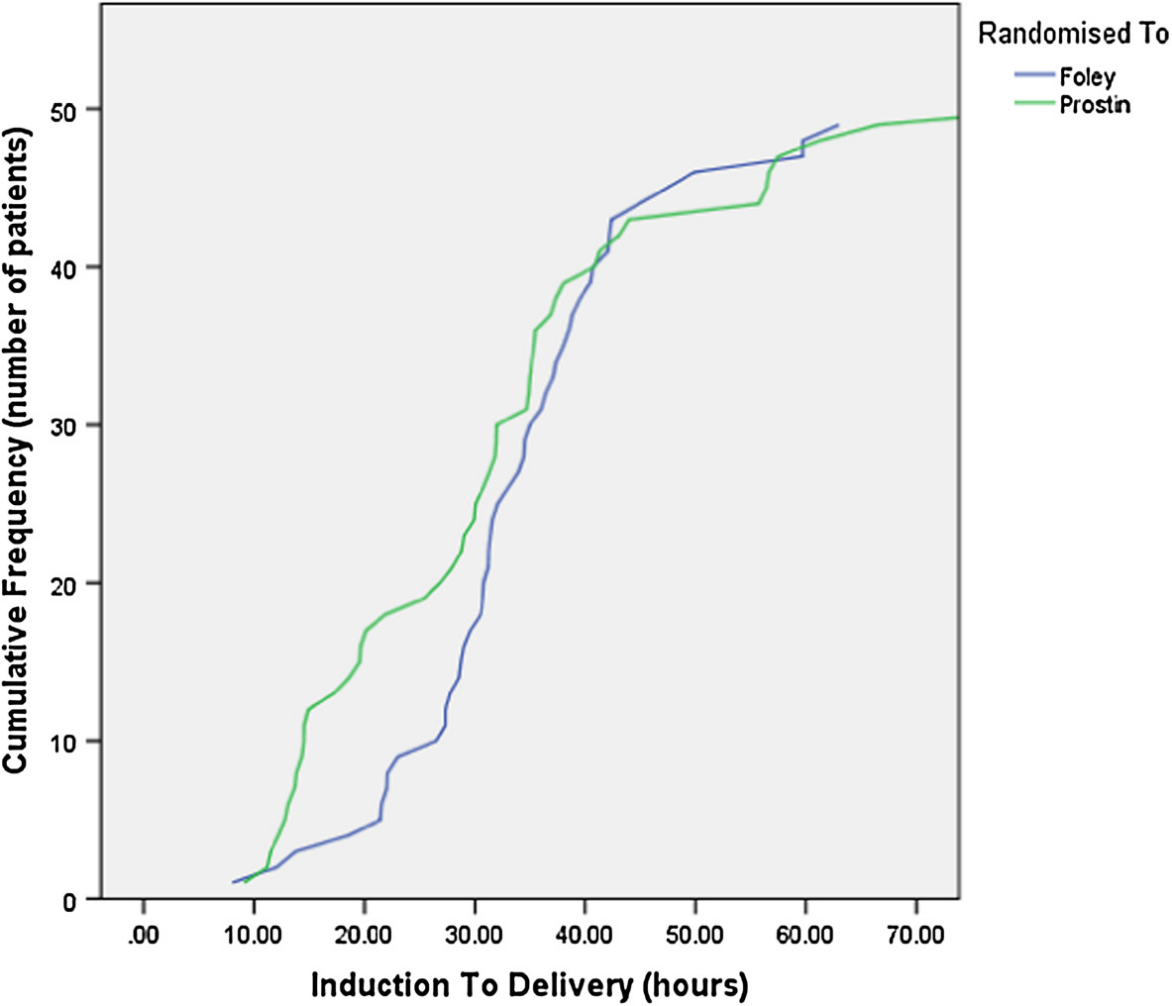
Induction to delivery interval comparison.

Pre-specified primary and secondary feasibility and clinical effectiveness outcomes are shown in Table 3. The OPC group spent significantly less time in hospital prior to birth, approximately 11 hours, however had a longer Birthing Unit stay, and their overall reduced inpatient stay (96 vs. 105 hours) was not statistically significant. For vaginal birth within 12 hours of Birthing Unit admission/transfer, IP was superior (53% vs. 28% OPC, p = .01), and need for oxytocin was greater in the OPC group. Total induction-to-delivery time did not differ between groups, 33.5 hr (+/−11.2) OPC Vs. 31.1 hr (+/−16.3) IP (Figure 2). Post-hoc analysis of Birthing Unit service loads found that the proportion of after 5 pm and after midnight deliveries was similar in the two groups, but more OPC women were admitted as scheduled at 0600–0900 (84% OPC vs. 43% IP, p = .001) and fewer between 5 pm and 6 am (8% vs. 33%, p = .002).

**Table 3:** Primary and secondary outcomes – feasibility and clinical effectiveness

**Primary and Secondary outcomes: Clinical effectiveness**	**Foley Catheter (N = 50)**	**PGE2 Gel (N = 51)**	**Odds ratio or mean difference (95% CI)**	**P-value**
**Primary outcomes**				
Vaginal delivery within 12 hours of admission to Birthing unit	14 (28%)	27 (53%)	**.35 (.15–.79)**	**.011**
Inpatient hours randomisation to birth	21.3 (+/−10.1)	32.4 (+/−16.9)	**−11.3 (−5.9 to −16.7)**	**<.001**
**Secondary outcomes**				
Mode of Delivery				.116
· **Vaginal Delivery**	*33 (66%)*	*36 (71%)*	.85 (.35-1.87)	.620
◦ Normal Vaginal Delivery	15 (30%)	25 (49%)		.051
◦ Instrumental Delivery	18 (36%)	11 (22%)	2.0 (0.85-4.9)	.109
· **Caesarean Section**	*17 (34%)*	*15 (29%)*		.620

Delivery Suite hours prior to birth	13.9+/−(7.5)	9.7+/−(5.1)		**.001**
Require Oxytocin	44 (88%)	30 (59%)	**5.1 (1.9–14.2)**	**.001**
· Duration - Oxytocin (Hrs)	11.6+/−(6.0)	9.0+/−(3.6)		**.035**
· Max Concentration – Oxytocin	53.7+/−(25.7)	45+/−(23.7)		.155
**Duration (Hrs)**				
· Cervical Ripening to Admission to Birthing unit	19.2+/−(7.0)	21.7+/−(19.2)		.245
· Cervical Ripening to Delivery Interval	33.5+/−(11/2)	31.1+/−(16.3)		.402
**Cervical ripening to vaginal delivery interval:**				
Vaginal Delivery within 24 Hours	6 (12%)	15 (29%)	**.33 (.12–.93)**	**.031**
Vaginal Delivery beyond 24 Hours	27 (54%)	21 (41%)		.197
**Outcome of cervical preparation**				.27
· Requirement for 3rd dose PGE2 (IP)		6 (12%)		
· Crossover to PGE2 for failed Foley (OPC)	2 (4%)			
**Total inpatient stay (hours)**	96+/−38	105+/−38	−9 (−24 to 7)	.267

Caesarean delivery rates were high in both groups (34% OPC Vs. 29% IP, NS). There were more instrumental deliveries in the OPC group although this did not reach statistical significance (Table 3).

Secondary safety outcomes are shown in Table 4. No differences in maternal morbidity were noted. Suspicious but not pathological CTG was significantly more common in OPC, but Apgar scores, neonatal admission rates, and cord gas values were similar between the groups.

**Table 4:** Safety outcomes

**Maternal intrapartum/postpartum complications**	**Foley Catheter (N = 50)**	**PGE2 Gel (N = 51)**	**P-value**
Intra-partum	7 (14%)	5 (10%)	
· Pyrexia	5	4	
· Other	2	1	
Immediate Post-partum	10 (20%)	12 (24%)	
· Post-partum haemorrhage	8	11	
· Other	2	1	
3rd degree perineal tear	2 (4%)	3 (6%)	.663
Other post-partum complication (requiring additional hospital treatment or extension of stay)	5 (10%)	9 (18%)	.266
**Neonatal intrapartum/postpartum complications**	**Foley Catheter (N = 50)**	**PGE2 Gel (N = 51)**	**P-value**
Abnormal CTG during Induction			
· Suspicious CTG	39 (78%)	28 (55%)	
· Pathological CTG	8 (16%)	5 (10%)	
Operative delivery for fetal concerns	23 (46%)	16 (31%)	.131
Hyperstimulation	0 (0%)	2 (4%)	.157
APGAR scores (mean and SD)			
· 1 minute	8.3+/−1.4	8.3+/−1.5	.873
· 5 minutes	8.9+/−0.7	8.9+/−0.5	.859
Cord Arterial pH <7.10	n = 29	n = 27	
	2 (7%)	4 (15%)	
Nursery Admission	8 (16%)	9 (18%)	
· Low pH or Respiratory Distress	5	8	
· Other	3	1	

Regarding feasibility of OPC, 45 women randomised to OPC received a catheter and 39 women (78%) went home with a catheter *in situ*. As shown in Figure 1, the predominant reason for being randomised to OPC but not receiving a catheter was being more dilated at attempted insertion than expected from the baseline VE, and the main reason women received a catheter but were not discharged home was concern regarding the post-insertion CTG. There were no cases of ‘tight os’ precluding catheter insertion. For discharged women average admission to discharge time was 4.4 hours (SD +/− 3.8). One discharged OPC patient represented (in labour) prior to scheduled readmission the following morning and two women sought phone advice.

When nulliparous and parous women were compared, all parous women had a vaginal birth (80% NVD, 20% instrumental) vs. 65% of nulliparous women (p = .02), and length of stay was significantly shorter in parous women (2.6 days vs. 4.4 days nulliparous, p < .01). When analysed by induction agent, there were no differences in trial outcomes between parous OPC and parous IP.

93 of 101 women completed the satisfaction survey (Table 5). The major differences between groups were in the amount of pain women felt during cervical ripening and their self-rated ability to relax, rest and sleep. OPC women were twice as likely to feel significant discomfort at the commencement of cervical preparation (55% vs. 29%, p = .01), but half as likely to feel significant discomfort throughout the cervical preparation phase (26% vs. 58%, p = .003). Sleep quantity was substantially greater in the OPC group (5.8 Vs. 3.4 hours, p < .001), which was only partially explained by a subgroup of IP entering labour overnight, and significantly fewer had safety concerns about their IOL (5% vs. 28%, p = .006).

**Table 5:** Satisfaction survey results

**Satisfaction Survey**	**Foley Catheter (N = 48)**	**PGE2 Gel (N = 45)**	**Odds ratio or mean difference (95% CI)**	**P-value**
Felt a lot of discomfort*				
· At insertion	26 (55%)	13 (29%)	**2.9 (1.2–6.9)**	**.014**
· 4–6 hours later	11 (23%)	16 (36%)		.18
· Overall cervical ripening	10 (26%) N = 39	25 (58%) N = 43	**0.25 (.10–.64)**	**.003**
Able to cope with discomfort*				
· At insertion	43 (92%)	39 (87%)		.914
· 4–6 hours later	37 (77%)	34 (76%)		.862
· Overall cervical ripening	37 (95%) N = 39	29 (67%) N = 43		**.002**
Would choose this method again#	31 (65%)	19 (42%)	**2.5 (1.1-5.8)**	**.031**
Took prescribed sleeping tablets	31 (65%)	27 (61%)		.648
Hours of sleep (before and/or after tablets)	5.8 (+/−2.0)	3.4 (+/−2.9)		**.001**
**Questions specific to women who actually received interventions as planned**	**Foley (N = 39)**	**Prostin (N = 43)**		
Able to relax*##	39 (100%)	28 (65%)	**1.5 (1.2–1.9)**	**.001**
Able to rest*##	39 (100%)	26 (61%)	**1.7 (1.3–2.1)**	**.001**
Worried the IOL not safe	2 (5%)	12 (28%)	**0.14 (.03–.67)**	**.006**
Embarrassed by catheter/gel	2 (5%)	2 (5%)		.920

* Agree or strongly agree.

# “Always” or “most times”.

## From time of insertion to time of Birthing Unit admission.

## Discussion

In this single-centre randomised trial of partially outpatient IOL with Foley catheter versus inpatient IOL with vaginal PGE2, we found that outpatient Foley catheter was feasible, of comparable overall clinical effectiveness, and acceptable to women compared to inpatient IOL with vaginal PGE2 gel.

Both important advantages and disadvantages of OPC vs. IP were noted. Consistent with its mechanical mode of action few in the OPC group laboured prior to their booked amniotomy, with consequently a greater need for oxytocin IOL and a longer Birthing Unit stay. Despite this, and consistent with previous studies of mechanical methods [3], Caesarean Section and induction to delivery intervals were similar between groups. As in prior Outpatient vs. Inpatient trials [7], OPC women spent significantly less time in hospital prior to the birth of their baby, but the overall decrease in length of stay failed to reach statistical significance.

**Figure 3:**
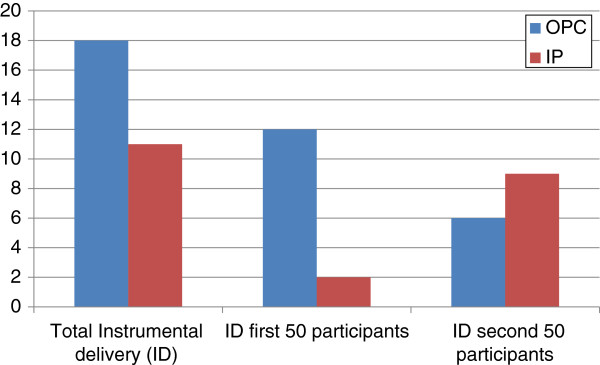
Comparison of Instrumental Delivery (ID) numbers.

Vaginal birth was achieved in only two-thirds of each group, did not differ between groups, and is consistent with recent local trial and population data on IOL outcome with unfavourable cervix (5, 12). The increased (borderline significance) instrumental delivery rate in OPC has not been noted in inpatient mechanical vs. chemical IOL trials [3], but was noted in a prior outpatient vs. inpatient PGE2 trial [11]. Although potentially related to increased oxytocin use and subsequent delivery for non-reassuring trace, the rate of oxytocin use remained constant during the trial while instrumental delivery in OPC decreased as the trial progressed (Figure 3). Therefore, initial increased instrumental delivery in OPC may have reflected the uncertainty of clinicians using OPC and a lower threshold for intervention, especially as the neonatal condition of these infants was similar to the overall cohort.

Regarding safety, both the issue of outpatient vs. inpatient IOL, and safety of the specific IOL method used, needs to be considered. Accordingly, catheter was chosen as our outpatient arm due to meta-analysis reporting lower rates of hyperstimulation than with Prostin [3], women in the OPC arm were not discharged unless post-insertion CTG was reassuring, and OPC women were advised to represent promptly if contracting regularly, membranes ruptured or they had any concerns. As expected hyperstimulation was not seen in the OPC group, however four OPC women (8%) remained inpatients due to CTG concerns post-insertion, compared to 13% of the outpatient arm in a recent report of outpatient vs. inpatient PGE2 [12]. One woman had a pathological trace at Birthing Unit admission after erroneously being told to remain at home when she called to report regular contractions. The baby delivered in good condition by Caesarean Section but with a cord pH of 7.03, underscoring the importance of advising women who commence labour after intervention to present for re-assessment. As only a small minority of women in our trial established in labour with OPC alone, this is likely to be an infrequent concern for OPC use.

The other major concern raised regarding safety of catheter IOL regards infectious morbidity, with a previous meta-analysis suggesting increased infectious morbidity with mechanical IOL [13]. These concerns were not borne out by the updated Cochrane review of mechanical methods of induction [3], although the number of studies reporting these outcomes remained small. Our trial clearly lacks the statistical power to address such rare but important safety outcomes, however it is hoped our data can be incorporated into future meta-analyses addressing these issues.

Maternal satisfaction with obstetric intervention, including induction of labour, is an under-studied area [14]. Only Pennell et al. [5] have reported on maternal satisfaction and pain scores with catheter vs. PGE2. Like their group, we found increased pain at insertion but decreased pain thereafter, and we have additionally shown superior rest and sleep scores for the catheter.

Acceptability of OPC was high, as reflected in the low rates of expressed anxiety about safety of the catheter, and strong agreement amongst the OPC group that they were able to rest/relax at home. Limitations of the satisfaction survey include its postpartum administration, meaning birth outcomes may have influenced patient responses, and the wish of many trial participants (in a hospital where outpatient IOL is not routinely available) to enter the OPC arm, possibly giving a bias towards favourable responses from OPC women.

The strengths of the study are its prospective, randomised design in a field (outpatient vs. inpatient induction) with few published trials. Like preceding outpatient vs. inpatient trials, the major limitations of the study are its small size and single-centre nature. Failure to reach initial planned recruitment targets is another major limitation, with only the lesser sample size for the second of our pre-specified primary outcomes reached. This was due to only two-thirds of the expected number of women presenting for cervical preparation during the study period at the study hospital, and a higher than expected percentage of screened but not recruited women. The study nevertheless represents the first randomised trial comparing outpatient Foley catheter with an inpatient prostaglandin preparation.

Another study limitation is difficulty in assessing the relative contributions of outpatient vs. inpatient IOL and mechanical vs. chemical IOL when the outpatient arm was mechanical and inpatient chemical. As PGE2 is the default local cervical preparation method, but potentially unsuitable for outpatient IOL given greater hyperstimulation rates than mechanical methods [3], the primary motivation for our study was exploring the role of the Foley catheter as a potentially feasible alternative to our current (chemical) inpatient standard. Although this inevitably reduces the research purity of the trial, as does the inclusion of (a) both nulliparous and parous women (b) a percentage of women with either maternal or fetal risk factors, we believe this pragmatic approach provided the best chance to assess the likely real-world role of OPC and is valid. Likewise, blinding of clinicians or patients to allocation was not practical, but apart from interpretation of traces and satisfaction survey responses, pre-specified outcomes were unlikely to be influenced by knowledge of allocation.

Finally, our unusual pre-specified primary outcomes are open to criticism. With hindsight, feasibility would have been better assessed using total inpatient hours rather than pre-delivery hours, and concerns regarding Birthing Services load, including overnight workload and its safety [15,16], assessed using a composite of hours in Birthing Services and the proportion of overnight transfers and births in Birthing Services. The pre-specified secondary outcomes, although underpowered, do include the standard IOL outcomes.

Although our study provides evidence of feasibility and patient acceptability of Foley catheter for partial outpatient IOL, there remains a need for larger studies, ideally at multiple sites, to demonstrate whether OPC yields equivalent birth outcomes (without compromising maternal and fetal safety) to inpatient methods, and whether the non-significant decreases in total inpatient hours noted in our trial and the Cochrane review [7] will be borne out. Parous women, who are under-represented in both outpatient vs. inpatient and mechanical vs. chemical trials, are a particular priority for study, as is economic analysis of outpatient vs. inpatient IOL.

## Conclusions

Where induction of labour for low-risk women with an unfavourable cervix is warranted, cervical ripening using a single-balloon catheter in the outpatient setting is feasible and acceptable to women compared to inpatient prostaglandin. Shorter antenatal hospital stay and maternal satisfaction with OPC needs to be balanced against a greater need for oxytocin IOL, and longer stay in Birthing Unit, when compared to IP. This choice of method for informed, low-risk women with reassuring baseline fetal status should be further evaluated within the context of prospective, multi-site research.

## Abbreviations

OPC: Outpatient foley catheter; IP: Inpatient prostin (PGE2); IOL: Induction of labour; FHR: Fetal heart rate; CTG: Cardiotocograph; VE: Vaginal examination.

## Competing interests

The authors declare that they have no competing interests.

## Authors’ contributions

AH designed the study, prepared the protocol, implemented the intervention, interpreted the data and drafted the article. AM contributed substantially to implementation of the intervention, data acquisition, and data interpretation, and critically revised the article. RR contributed substantially to implementation of the intervention, data acquisition, and revision of the article. KG contributed substantially to implementation of the intervention, data acquisition, and revision of the article. ST contributed substantially to study design and critical revision of the article. AW contributed substantially to data analysis/interpretation and critical revision of the article. DC designed the original study concept, and contributed substantially to study design, implementation of the intervention, and preparation and revision of the article. All authors read and approved the final manuscript.

## Pre-publication history

The pre-publication history for this paper can be accessed here:

http://www.biomedcentral.com/1471-2393/13/25/prepub

## Supplementary Material

Additional file 1 **Satisfaction Survey.doc. **Template of Patient Satisfaction Questionnaire used in the study. Click here for file
