# Supplementary material for: Outpatient Foley catheter versus inpatient prostaglandin E2 gel for induction of labour: a randomised trial
Source: BMC Pregnancy Childbirth. 2013 Jan 29;13:25. doi: 10.1186/1471-2393-13-25 (PMC3564932; doi:10.1186/1471-2393-13-25)
Supplement: Additional file 1 — Satisfaction Survey.doc. Template of Patient Satisfaction Questionnaire used in the study. [file 1471-2393-13-25-S1.doc]

**A comparison of Outpatient Foley’s Catheter and Inpatient**

**Prostaglandin Gel for Cervical Ripening in Induction of Labour:**

# Patient Questionnaire

You are invited to fill in this questionnaire to tell us about the care you received at the Royal Hospital for Women when you had your labour induced. The results of this survey will help the managers to see where the system is working well and where things need to be done differently. The data will also be included as part of the research we are currently doing at the Royal Hospital for Women to tell us what methods of induction women prefer to be offered. All responses will be kept **strictly confidential** by both research and administrative staff collating this data. The survey will take about **15 minutes** to complete.

**Part A**

*For each of the following statements please circle*

*the number which shows how you felt about your induction*

Some- Most

Never Rarely times times Always

A I felt anxious about being part of the study 1 2 3 4 5

B I felt in control 1 2 3 4 5

C I understood what was happening 1 2 3 4 5

D I felt relaxed 1 2 3 4 5

E Everything made sense 1 2 3 4 5

F I was given clear information 1 2 3 4 5

G I felt comfortable with my choice about my care 1 2 3 4 5

H I had access to information about the types of

induction available 1 2 3 4 5

I I had easy access to information about what to do 1 2 3 4 5

J I found the induction process uncomfortable 1 2 3 4 5

K I was worried about when my labour would begin 1 2 3 4 5

L I would choose this method of induction next time 1 2 3 4 5

M I would recommend this method of induction 1 2 3 4 5

to other women

**Part B** *(please circle answer)*

**1) Thinking about** **when the catheter or gel was first inserted how much do you agree or disagree with the following:**

Strongly Strongly

agree Agree Unsure Disagree disagree

I felt a lot of discomfort 1 2 3 4 5 I was able to cope with the discomfort 1 2 3 4 5 I felt tense and anxious during the insertion 1 2 3 4 5

I felt anxious that the induction wouldn’t work 1 2 3 4 5

**2) Thinking about** **4-6 hours *after* the catheter or gel was first inserted how much do you agree or disagree with the following:**

Not applicable (baby already delivered) Y/N **If Y, go to question 4**

Strongly Strongly

agree Agree Unsure Disagree disagree

I felt a lot of discomfort 1 2 3 4 5 I was able to cope with the discomfort 1 2 3 4 5 I felt tense and anxious 1 2 3 4 5

I felt anxious that the induction wouldn’t work 1 2 3 4 5

I felt that my labour had started 1 2 3 4 5

**3) Thinking about** **10-12 hours *after* the catheter or gel was first inserted how much do you agree or disagree with the following:**

Not applicable (baby already delivered) Y/N **If Y, go to question 4**

Strongly Strongly

agree Agree Unsure Disagree disagree

I felt a lot of discomfort 1 2 3 4 5 I was able to cope with the discomfort 1 2 3 4 5 I felt tense and anxious 1 2 3 4 5

I felt anxious that the induction wouldn’t work 1 2 3 4 5

I felt that my labour had started 1 2 3 4 5

1. **Thinking about the time from when the gel or catheter was first inserted until the time you were admitted to Delivery Suite or Birth Centre, please estimate how many hours of sleep you had** *(circle one response in each line)*:
2. *Without/before tablets for pain relief or to help you sleep*

No sleep <1 hour 1-2hours 2-4 hours 4-6 hours >6 hours

1. *After taking tablets for pain relief or to help you sleep*

No sleep <1 hour 1-2hours 2-4 hours 4-6 hours >6 hours

Not applicable (did not take tablets)

**5) Thinking about** **the induction of labour from the time you were admitted to Delivery Suite or Birth Centre to the time the baby was born how much do you agree or disagree with the following:**

Not applicable (baby delivered before getting to Delivery Suite or Birth Centre)

Y/N **If Y, go to question 6**

Strongly Strongly

agree Agree Unsure Disagree disagree

I felt a lot of discomfort 1 2 3 4 5 I was able to cope with the discomfort 1 2 3 4 5 I felt tense and anxious 1 2 3 4 5

I felt anxious that the induction wouldn’t work 1 2 3 4 5

I felt that my labour had started 1 2 3 4 5

1. **Before you went into labour or had your baby did you have any particular worries about labour and birth?** *(circle one number in each line)*

Very Quite Not very Not at all N/A worried worried worried worried

Not knowing if the induction would work 1 2 3 4 5

Not knowing when I would go into labour 1 2 3 4 5

Getting to the hospital in time 1 2 3 4 5

Having a long labour 1 2 3 4 5

Pain and discomfort in labour 1 2 3 4 5

Getting effective pain relief 1 2 3 4 5

Not knowing how long labour would take 1 2 3 4 5 Having forceps or a ‘ventouse’ 1 2 3 4 5

Needing a caesarean section 1 2 3 4 5

Being in labour a long time 1 2 3 4 5

Other (please specify)______________________________________________________________

**Part C: Foley catheter group**

**1) Thinking about** **the time from when you went home until the time you came back in to hospital, how much do you agree or disagree with the following:**

Not applicable (did not get to go home) Y/N

Strongly Strongly

agree Agree Unsure Disagree disagree

I felt a lot of discomfort 1 2 3 4 5 I was able to cope with the discomfort 1 2 3 4 5 I felt anxious about going home 1 2 3 4 5

While at home I felt anxious being at home 1 2 3 4 5

rather than in hospital

I was able to relax at home 1 2 3 4 5 I was able to rest at home 1 2 3 4 5 I had good family support at home 1 2 3 4 5

I had easy access to information from the hospital 1 2 3 4 5

I was worried it might not be safe to be at home 1 2 3 4 5 I would have preferred to be in the other study group 1 2 3 4 5 I felt embarrassed by the catheter 1 2 3 4 5

**2) If you had another induction would you book at the Royal Hospital for Women again (please circle)?**

Yes No

If not, why not?

**________________________________________________________________________________**

**________________________________________________________________________________**

**________________________________________________________________________________**

**________________________________________________________________________________**

**3) Is there anything else you would like to tell us?**

**________________________________________________________________________________**

**________________________________________________________________________________**

**________________________________________________________________________________**

**________________________________________________________________________________**

**________________________________________________________________________________**

**THANK YOU for taking the time to complete this questionnaire**

If you leave hospital before our study researcher returns to collect the questionnaire please seal it in the envelope provided and leave it with the staff member looking after you

**Part C: Prostin group**

**1) Thinking about** **the time from when the first dose of gel was inserted on the Antenatal Ward to the time you went to Delivery Suite or Birth Centre how much do you agree or disagree with the following:**

Strongly Strongly

agree Agree Unsure Disagree disagree

I felt a lot of discomfort 1 2 3 4 5 I was able to cope with the discomfort 1 2 3 4 5 I felt anxious about being in hospital 1 2 3 4 5

I was able to relax on the Antenatal Ward 1 2 3 4 5 I was able to rest on the Antenatal Ward 1 2 3 4 5 I had good family support in hospital 1 2 3 4 5

I had easy access to information from the staff 1 2 3 4 5

I was worried the induction might not be safe 1 2 3 4 5 I would have preferred to be in the other study group 1 2 3 4 5 I felt embarrassed by the gel 1 2 3 4 5

**2) If you had another induction would you book at the Royal Hospital for Women again (please circle)?**

Yes No

If not, why not?

**________________________________________________________________________________**

**________________________________________________________________________________**

**________________________________________________________________________________**

**________________________________________________________________________________**

**3) Is there anything else you would like to tell us?**

**________________________________________________________________________________**

**________________________________________________________________________________**

**________________________________________________________________________________**

**________________________________________________________________________________**

**________________________________________________________________________________**

**THANK YOU for taking the time to complete this questionnaire**

If you leave hospital before our study researcher returns to collect the questionnaire please seal it in the envelope provided and leave it with the staff member looking after you

Note to researchers: There are two separate Part C sections, one for the Foley Catheter group and one for the Prostin group. Ensure that each study subject receives only the Foley Catheter *or* Prostin page as applicable, not both. Each woman should have only a four page questionnaire to fill out.
